# RIP1-Dependent and Independent Effects of Necrostatin-1 in Necrosis and T Cell Activation

**DOI:** 10.1371/journal.pone.0023209

**Published:** 2011-08-10

**Authors:** YoungSik Cho, Thomas McQuade, Haibing Zhang, Jianke Zhang, Francis Ka-Ming Chan

**Affiliations:** 1 Department of Pathology, Program in Immunology and Virology, The University of Massachusetts Medical School, Worcester, Massachusetts, United States of America; 2 Department of Microbiology and Immunology, Kimmel Cancer Center, Thomas Jefferson University, Philadelphia, Pennsylvania, United States of America; 3 UMass Diabetes and Endocrinology Research Center, The University of Massachusetts Medical School, Worcester, Massachusetts, United States of America; Oklahoma Medical Research Foundation, United States of America

## Abstract

**Background:**

Programmed necrosis/necroptosis is an emerging form of cell death that plays important roles in mammalian development and the immune system. The pro-necrotic kinases in the receptor interacting protein (RIP) family are crucial mediators of programmed necrosis. Recent advances in necrosis research have been greatly aided by the identification of chemical inhibitors that block programmed necrosis. Necrostatin-1 (Nec-1) and its derivatives were previously shown to target the pro-necrotic kinase RIP1/RIPK1. The protective effect conferred by Nec-1 and its derivatives in many experimental model systems was often attributed to the inhibition of RIP1 function.

**Methodology/Principal Findings:**

We compared the effect of Nec-1 and siRNA-mediated silencing of RIP1 in the murine fibrosarcoma cell line L929. Treatment of L929 cells with the pan-caspase inhibitor zVAD-fmk or exogenous TNF induces necrosis. Strikingly, we found that siRNA-mediated silencing of RIP1 inhibited zVAD-fmk induced necrosis, but not TNF-induced necrosis. TNF-induced cell death in RIP1 knocked down L929 cells was inhibited by Nec-1, but not the caspase inhibitor zVAD-fmk. We found that PKA-C§ expression, but not Jnk or Erk activation, was moderately inhibited by Nec-1. Moreover, we found that Nec-1 inhibits proximal T cell receptor signaling independent of RIP1, leading to inhibition of T cell proliferation.

**Conclusions/Significance:**

Our results reveal that besides RIP1, Nec-1 also targets other factors crucial for necrosis induction in L929 cells. In addition, high doses of Nec-1 inhibit other signal transduction pathways such as that for T cell receptor activation. These results highlight the importance to independently validate results obtained using Nec-1 with other approaches such as siRNA-mediated gene silencing. We propose that some of the previous published results obtained using Nec-1 should be re-evaluated in light of our findings.

## Introduction

Cell death by necrosis is often associated with inflammation. Although necrosis was once thought to be an un-regulated form of cell injury due to trauma, recent evidence indicates that necrosis is a highly regulated process that involves a dedicated molecular circuitry [1], [2]. Specifically, necrosis is the dominant response when cells are stimulated with TNF-like death cytokines in the presence of caspase inhibitors [1]. This form of necrosis, often referred to as programmed necrosis/necroptosis [3], is characterized by organelle and cell swelling and eventually plasma membrane rupture. In necrosis, the loss of plasma membrane integrity often coincides or precedes the exposure of phosphatidyl serine [4]. This morphological signature of necrosis distinguishes it from apoptosis, in which the early exposure of phosphatidyl serine and other “eat-me” signals prompt their clearance by professional phagocytes before membrane leakage and release of inflammatory cellular adjuvants [5]. Thus, necrosis is a more inflammatory form of cell death than apoptosis.

Recent work shows that the receptor interacting protein (RIP) kinases RIP1 and RIP3 play essential roles in TNF-induced necrosis [6], [7], [8]. In addition to TNF-like death cytokines, RIP1/RIP3-dependent necrosis also contributes to the abortive embryonic development and impaired T lymphocyte proliferation of FADD^−/−^ and caspase-8^−/−^ mice [9], [10], [11], [12], [13]. Furthermore, RIP1/RIP3-dependent necrosis has been shown to mediate cerulein-induced pancreatitis [7], [8], retinal detachment induced photoreceptor necrosis [14], ischemia-induced brain injury [15], [16] and myocardial infarction [17].

Necrostatin-1 (Nec-1) is a small molecule inhibitor originally identified in a chemical library screen as a potent inhibitor of TNF-induced necrosis [15]. Subsequent studies show that Nec-1 specifically inhibits the pro-necrotic kinase function of RIP1 [18], but has no effects on another pro-necrotic kinase RIP3 [6]. Moreover, Nec-1 does not interfere with TNF-induced NF-kappa B activation [15], an effect that is also driven by RIP1. Because of its relatively specific effect against the pro-necrotic activity of RIP1, Nec-1 has become a popular tool to probe the role of necrosis in different experimental models of cell injury.

In this report, we show that Nec-1 inhibits necrosis in a RIP1-independent manner in L929 cells. In addition, at doses that are commonly used in many studies, Nec-1 impairs the sustained phosphorylation of linker for activation of T cells (LAT), a crucial adaptor in proximal T cell receptor (TCR) signaling. As a result, Nec-1 treated cells failed to expand and proliferate in response to TCR stimulation. These results reveal that Nec-1 exhibits RIP1-dependent and independent effects in necrosis and other signal transduction processes. Thus, care should be taken in interpreting results derived from using this inhibitor.

## Materials and Methods

### Ethics Statement

All studies involving animals were approved by the University of Massachusetts Institutional Animal Care and Use Committee (IACUC) (protocol #1396). Animals were maintained in accordance with the Guide for the Care and Use of Laboratory Animals (Institute of Laboratory Animal Resources, 1996).

### Cell death assays

L929 and NIH 3T3 cells (ATCC, Manassas, VA, USA) were cultured in Dulbecco's modified Eagle medium. Jurkat cells [19], [20] and primary T cells isolated from wild type and FADD^−/−^ mice [21] were cultured in RPMI 1640 medium. All media were supplemented with 10% fetal bovine serum, 100 units/ml penicillin, 100 µg/ml streptomycin, 30 µg/ml L-glutamine, and 60 µM 2-mercaptoethanol. For cell death assays, cells were seeded at 5,000–10,000 cells per well on 96-well microtiter plates and stimulated as indicated. For L929 and NIH 3T3 cells, 10 ng/ml of recombinant mouse TNF was used (Invitrogen, Carlsbad, CA, USA). For NIH 3T3 cells were pre-treated with 0.5 µg/ml cycloheximide and 10 µM zVAD-fmk (Axxora, San Diego, CA, USA) for one hour before TNF stimulation. For Jurkat cells, necrosis was induced by pretreatment with 20 µM zVAD-fmk for 1 hour and 100 ng/ml human TNF (Invitrogen, Carlsbad, CA, USA). MAP kinase inhibitors (Enzo Life Sciences, Farmingdale, NY, USA) were used at the following concentrations: Erk inhibitor U0126 (50 µM), Jnk inhibitor II (30 µM), p38 inhibitor SB203580 (50 µM). Cell death was determined 6–14 hours post-TNF treatment by CellTiter aqueous non-radioactive cell proliferation assay (MTS) (Promega, Madison, WI, USA). In some experiments, we confirmed cell death by flow cytometry using propidium iodide uptake as an indication of cell death (data not shown).

### siRNA transfection

Short oligonucleotide RNAs (27-mer siRNA) were purchased from IDT technologies (Coralville, IA, USA). For transfection of siRNA to L929 cells or NIH 3T3 cells, 300,000 cells were seeded on 6-well plate and transfected with 20 nM siRNA and 12 µl HiPerfect reagent (Qiagen, Valencia, CA, USA). Thirty-six hours after transfection, cells were replated on 96-well plate (5,000 cells per well) overnight before stimulation with recombinant mouse TNF. siRNA sequences used are: RIP1 #1 (5′-gaggauauucucaggcuucagguccuu-3′), RIP1 #2 (5′-ccuucguuuccuuuccuccucucuguu-3′), RIP3 (5′-aagauuaaccauagccuucaccuccca-3′).

### Western blot

Cell lysates or immune complexes were analyzed by standard SDS-PAGE. After transfer to nitrocellulose membranes, Western blots were performed with antibodies to RIP1, §-actin, PARP-1 (BD Pharmingen, San Diego, CA, USA), RIP3 (ProSci, Poway, CA, USA), Erk, Jnk, PKA substrate (Cell Signaling, Danvers, MA, USA), PKA, LAT (Santa Cruz, Santa Cruz, CA, USA), and phospho-tyrosine (Millipore, Bellerica, MA, USA).

### Fluorogenic caspase 3 activity assay

Fifty micrograms of cell extracts were diluted in 200 µl of iTFB buffer (10% sucrose, 30 µM HEPES [pH 7.4], 10 µM CaCl_2_ and 5 µM DTT) containing 25 µM of N-acetyl-Asp-Glu-Val-Asp-7-amino-4-methylcoumarin (Ac-DEVD-AMC) (Axxora, San Diego, CA, USA). The release of fluorescent AMC was measured in a FLUOSTAR multi-well plate reader at 340 nm for excitation and 450 nm for emission over 3 hours. The rate of conversion was calculated by including 25 µM of free AMC as control.

### T cell activation

CD3^+^ T cells were purified from the spleens of 10–12 week old C57BL/6 mice using the Dynal negative T cell isolation kit (Invitrogen). Flow cytometry shows that the resulting cells were >98% CD3^+^ (data not shown). Unless otherwise stated, purified T cells were stimulated with plate-bound 1 µg/ml anti-CD3 and 200 ng/ml anti-CD28 antibodies (eBioscience, San Diego, CA, USA). For thymidine incorporation, 1 µCi of [^3^H]-thymidine was added to the cells on day 3 for 18 hours before harvesting. For CellTracer Violet dilution assay, cells were loaded with 5 µM CellTracer Violet as per manufacturer's protocol (Invitrogen). Three days after stimulation, dilution of the dye was determined on a BD LSR2 flow cytometer and analyzed with Flowjo software (Treestar, Ashland, OR, USA). For Western blot and immunoprecipitations of T cell lysates, purified T cells were stimulated with 3 µg/ml biotinylated anti-CD3 antibody and 0.5 µg/ml streptavidin for the indicated times before lysis in buffer containing 150 mM NaCl, 20 mM Tris.Cl [pH 7.4], 0.2% NP-40, 1 mM EDTA, 3 mM NaF, 1 mM β-glycerophosphate, 1 mM sodium orthovanadate, 10% glycerol, 1× protease inhibitor cocktail (Roche), and 1× phosphatase inhibitor cocktail (Sigma).

## Results

**Figure 1:**
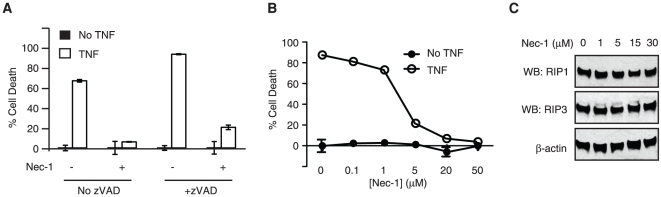
Nec-1 inhibits necrosis in L929 cells in a dose-dependent manner. (A) Nec-1 inhibits TNF-induced necrosis in L929 cells. L929 cells were treated with 10 µM zVAD-fmk, 20 µM Nec-1 and 10 ng/ml mouse TNF (mTNF) as indicated. Cell death was determined by MTS assay as described in materials and methods. (B) L929 cells were treated with the indicated doses of Nec-1, followed by stimulation with 10 ng/ml mTNF. Cell Death was analyzed as in (A). (C) Nec-1 did not alter RIP1 or RIP3 protein expression in L929 cells.

The murine fibrosarcoma L929 cells are unique in that they readily undergo necrosis in response to TNF [22]. TNF-induced necrosis in L929 cells did not require caspase inhibition, although the caspase inhibitor zVAD-fmk did further enhance TNF-induced necrosis (Fig. 1A). Regardless of whether zVAD-fmk was used, Nec-1 inhibited TNF-induced necrosis in L929 cells in a dose-dependent manner, reaching maximal inhibition at 20 µM or higher (Fig. 1B and data not shown). This dose of Nec-1 (20–50 µM) was commonly used in many publications [9], [15], [18], [23]. Although Nec-1 has been reported to inhibit the expression of ectopically expressed RIP1 in HEK293T cells [6], it did not affect endogenous RIP1 expression in L929 cells (Fig. 1C). These results are consistent with previous results that Nec-1 is an allosteric inhibitor of RIP1 kinase function [18].

**Figure 2:**
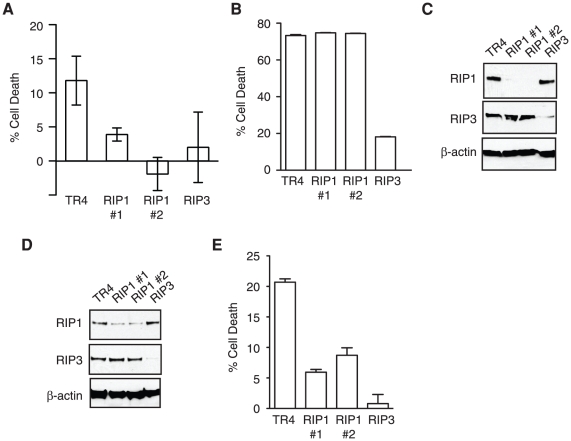
Nec-1 inhibits TNF-induced necrosis independent of RIP1 in L929 cells. (A–C) RIP1 is required for zVAD-fmk, but not TNF-induced necrosis in L929 cells. (A) L929 cells were transfected with the indicated siRNA oligonucleotides with HiPerfect (Qiagen). TR4 is the control siRNA against TRAIL-R4 [36]. Cells were treated with (A) 50 µM zVAD-fmk or (B) 10 ng/ml mTNF and cell death was measured by MTS assay. (C) Western blot shows RIP1 and RIP3 expression in cells transfected with the indicated siRNAs. (D–E) RIP1 is required for TNF-induced necrosis in 3T3 fibroblasts. NIH 3T3 fibroblasts were transfected with the indicated siRNA. (D) Expression of RIP1, RIP3 and ß-actin in cells transfected with the indicated siRNA was determined by Western blot. (E) Necrosis was induced with 10 µM zVAD-fmk, 0.5 µg/ml cycloheximide and 10 ng/ml mTNF for 20 hours. Cell death was assessed as in (A).

In addition to exogenous TNF, treatment with the caspase inhibitor zVAD-fmk alone also causes necrosis in L929 cells [24]. zVAD-fmk induced necrosis in L929 cells is mediated by autocrine production of TNF. It exhibits a slower kinetics than TNF-induced necrosis and is optimally induced at high cell density and small volume of the culture medium [25]. Under these conditions, siRNA-mediated silencing of RIP1 by two different siRNAs efficiently protected L929 cells from zVAD-fmk induced necrosis (Fig. 2A). This is consistent with previous reports that RIP1 is required for necrosis induced by zVAD-fmk [24]. Strikingly, siRNA-mediated silencing of RIP1 had no effects on TNF-induced necrosis in L929 cells (Fig. 2B). Western blot analysis shows that the lack of protection against TNF-induced necrosis in L929 cells was not due to inefficient silencing of RIP1 expression (Fig. 2C). By contrast, similar siRNA-mediated silencing of RIP1 in NIH 3T3 fibroblasts, which was less efficient compared with that achieved in L929 cells (Fig. 2D), effectively inhibited TNF-induced necrosis (Fig. 2E). Furthermore, siRNA-mediated silencing of another essential programmed necrosis mediator, RIP3, effectively inhibited zVAD-fmk and TNF-induced necrosis in L929 cells (Fig. 2A–B), and TNF-induced necrosis in NIH 3T3 cells (Fig. 2E). Our results are consistent with those of Hitomi et al, which show that siRNA knock-down of RIP1 in L929 cells exhibited disparate effects on necrosis induced by zVAD-fmk or TNF [26].

**Figure 3:**
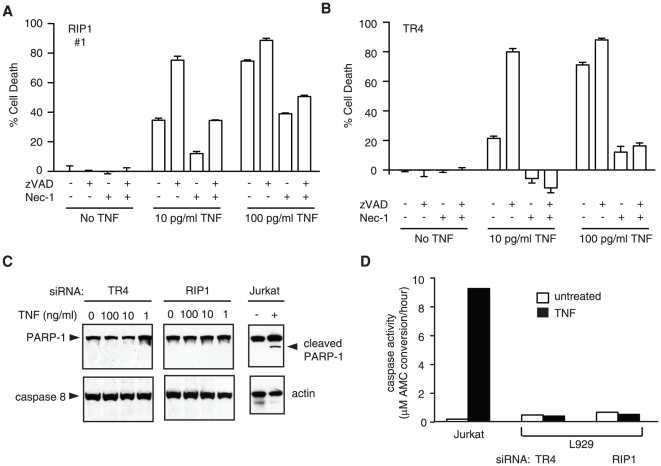
Knock-down of RIP1 expression in L929 cells did not result in caspase-dependent apoptosis. L929 cells were transfected with siRNA against (A) RIP1 or (B) TR4. Transfected cells were treated with zVAD-fmk, Nec-1 and TNF as indicated. Cell death was determined by MTS assay. (C) L929 cells transfected with the indicated siRNA or Jurkat 4E3 cells were treated with TNF. PARP-1 cleavage was determined by Western blot. (D) Cell lysates from (C) were tested for active caspase 3 as measured by cleavage of the substrate DEVD-AMC. Release of AMC was determined by increases in AMC fluorescence as described in materials and methods.

Inhibition of the pro-survival transcription factor NF-kappa B sensitizes cells to TNF-induced apoptosis. Because RIP1 is required for activation of NF-kappa B, knock-down of RIP1 could have sensitized L929 cells to TNF-induced apoptosis. In addition, RIP1 competes with TRADD for binding to TNFR1 [27]. Since TRADD promotes FADD recruitment and apoptosis, loss of RIP1 could activate caspase-dependent apoptosis. To evaluate this possibility, we treated RIP1-silenced L929 cells with the pan-caspase inhibitor zVAD-fmk and/or the RIP1 inhibitor Nec-1. Consistent with previous results, zVAD-fmk increased TNF-induced necrosis in RIP1 siRNA (Fig. 3A) and control siRNA transfected cells (Fig. 3B). In contrast, Nec-1 effectively inhibited TNF-induced necrosis in RIP1 siRNA (Fig. 3A) and control siRNA transfected cells (Fig. 3B). Nec-1 also inhibited cell death induced by TNF and zVAD-fmk in RIP1 siRNA transfected cells (Fig. 3A). Interestingly, residual death could be detected in RIP1 knock-down L929 cells treated with TNF, zVAD-fmk, and Nec-1, suggesting the possibility of caspase- and RIP1-independent cell death under these conditions. PARP-1 cleavage by caspases was not detected in RIP1 siRNA transfected L929 cells, but was readily detected in TNF-treated Jurkat cells (Fig. 3C). Furthermore, caspase activity as measured by cleavage of the fluorogenic peptide substrate DEVD-AMC was not detected in RIP1 or control siRNA transfected L929 cells, but was readily detected in TNF-treated Jurkat cells (Fig. 3D). Thus, TNF-induced necrosis in L929 cells is RIP1-independent and yet inhibitable by Nec-1.

**Figure 4:**
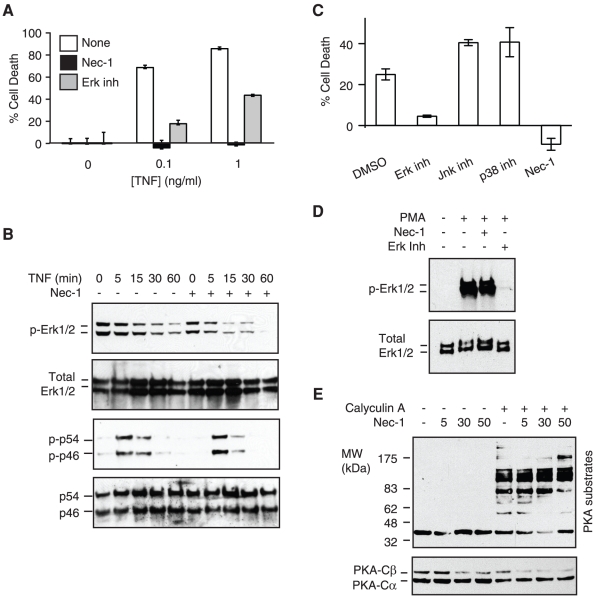
Effects of Nec-1 on Jnk, Erk and PKA-Cβ. (A) L929 cells were treated with TNF in the presence of 20 µM Nec-1 or 50 µM of the Erk inhibitor U0126. Cell death was determined by MTS assay. (B) The effect of Nec-1 on Erk or Jnk phosphorylation. L929 cells treated with 10 ng/ml of mTNF in the presence or absence of 30 µM Nec-1 were analyzed for Erk activation (p-p54 and p-p46) or Jnk activation by Western blot. (C) Erk inhibitor U0126 and Nec-1 blocks TNF-induced necrosis in Jurkat cells. Jurkat 4E3 cells [20] were treated with Erk inhibitor (50 µM U0126), Jnk inhibitor (30 µM Jnk inhibitor II), p38 inhibitor (50 µM SB203580), or 10 µM Nec-1 and stimulated with 100 ng/ml recombinant human TNF plus 10 µM zVAD-fmk for 14 hours. Cell death was determined by MTS assay. (D) Jurkat 4E3 cells were stimulated with 10 ng/ml PMA for 10 minutes in the presence of 30 µM Nec-1 or 50 µM U0126. Phospho-Erk was examined by Western blot as indicated. (E) Jurkat 4E3 cells were treated with the indicated doses of Nec-1 and 100 nM calyculin A. The expression of the two different PKA isoforms and phosphorylation of protein kinase A substrates was determined by Western blot with specific μantibodies as described in materials and methods.

A recent report indicates that Nec-1 inhibits Erk activation in TNF and Smac mimetic treated L929 cells [28]. Although the authors found that Erk inhibition did not protect L929 cells against TNF/Smac mimetic induced necrosis [28], we found that the Erk inhibitor U0126 inhibited TNF-induced necrosis in L929 cells, albeit at a lower level than that by Nec-1 (Fig. 4A). Surprisingly, phospho-Erk was readily detected in untreated L929 cells, and TNF treatment led to reduction in phosphorylated Erk (Fig. 4B, top two panels). By contrast, TNF-induced a transient phosphorylation of Jnk1/2 (Fig. 4B, p54 and p46, bottom two panels). While Nec-1 had no effects on Jnk activation, the reduction of Erk phosphorylation was more prominent in the presence of Nec-1 (Fig. 4B). To more definitively determine the effect of Nec-1 on Erk, we tested the effect of Nec-1 in Jurkat cells. TNF and zVAD-fmk induced necrosis in Jurkat 4E3 cells [19], [20], which was effectively inhibited by the Erk inhibitor U0126 or Nec-1 (Fig. 4C). By contrast, inhibition of p38 or Jnk had no effects on TNF/zVAD-fmk induced necrosis in Jurkat 4E3 cells (Fig. 4C). TNF stimulation did not induce detectable level of Erk phosphorylation in Jurkat 4E3 cells (data not shown). To evaluate whether Nec-1 inhibits Erk activation in Jurkat cells, we treated the cells with phorbol myristate acetate (PMA), which strongly induced Erk phosphorylation. We found that Nec-1 did not inhibit on Erk phosphorylation in Jurkat 4E3 cells under these conditions (Fig. 4D). Thus, the effect of Nec-1 on Erk phosphorylation in L929 cells is likely due to inhibition of RIP1, which is required for downstream Erk activation in certain cells [29].

A recent report indicates that Nec-1 could inhibit PKA activity in vitro [30]. Interestingly, we previously identified PKA-C§ as a putative necrosis regulator in a RNA interference screen [6]. We therefore tested whether Nec-1 might affect PKA functions. Treatment of Jurkat 4E3 cells with Nec-1 led to reduced expression of PKA-C§ in a dose-dependent manner (Fig. 4E, lower panel). Although Nec-1 had minimal effects on calyculin A induced phosphorylation of PKA substrates (Fig. 4E, top panel), this could be due to the compensatory effects of PKA-Cα and other calyculin A sensitive kinases. Thus, PKA-C§ might be one of the RIP independent targets of Nec-1.

**Figure 5:**
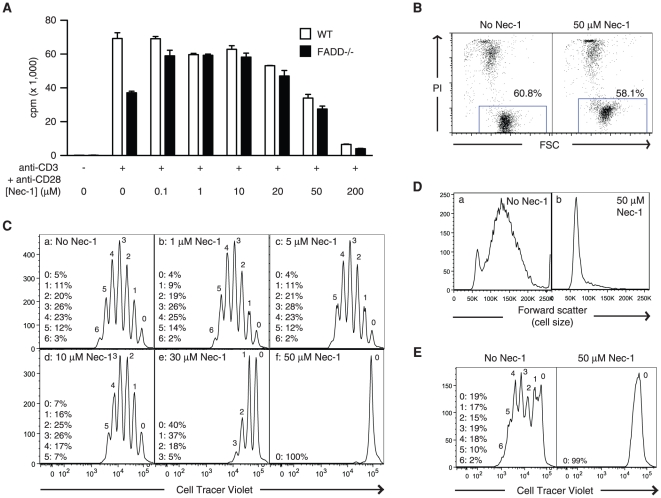
High doses of Nec-1 inhibit T cell proliferation. (A) Low dose Nec-1 inhibits TCR-induced necrosis, but high dose Nec-1 inhibits T cell proliferation. Purified T cells from wild type or FADD^−/−^ mice were stimulated with plate-bound anti-CD3, anti-CD28 and increasing doses of Nec-1. Three days later, cell proliferation was measured by incorporation of [^3^H]-thymidine. Results shown are mean ± SEM. (B) Nec-1 did not compromised T cell viability. Naïve CD3^+^ T cells were purified from the spleen of wild type C57BL/6 mice and incubated at 37°C for 24 hours in the presence or absence of 50 µM Nec-1. Viability of the cells was determined by flow cytometry using propidium iodide (PI) uptake as an indication of cell death. Note that Nec-1 increased the baseline fluorescence of the T cells. (C) Nec-1 inhibits T cell division. Purified CD3^+^ primary T cells were labeled with CellTracer Violet fluorescent dye and stimulated with 1 µg/ml plate-bound anti-CD3 and 200 ng/ml anti-CD28 antibodies. Three days later, cell division was analyzed using a BD LSR2 flow cytometer. The numbers above each peak represent the number of cell division the cells had undergone. The numbers on the left represent the percentages of cells in each peak. (D) Nec-1 inhibits T cell blast formation. Purified CD3^+^ T cells were similarly activated as in (C). Three days later, formation of T cell blast as measured by forward scatter was determined by flow cytometry. (E) FADD^−/−^RIP1^−/−^ DKO T cells stimulated with plate-bound anti-CD3 and anti-CD28 antibodies in the absence or presence of Nec-1 were measured for cell proliferation as in (C).

In addition to inhibiting TNF-induced necrosis, Nec-1 has also been shown to rescue necrosis in caspase-8^−/−^ [9] and FADD^−/−^ T cells [23] in response to T cell receptor (TCR) stimulation. Indeed, low doses of Nec-1 (<1 µM) were sufficient to inhibit necrosis and rescue defective proliferation of FADD^−/−^ T cells (Fig. 5A) [31]. Strikingly, we found that higher doses of Nec-1 (>20 µM) led to reduced proliferation of wild type and FADD^−/−^ T cells in a dose-dependent manner (Fig. 5A). The inhibition of T cell proliferation by high doses of Nec-1 might be due to inhibition of cell proliferation or increased cytotoxicity. Incubation of naïve T cells *in vitro* without stimulation led to cell death by neglect. Consistently, the viability of untreated T cells 24 hours later was reduced to ∼60% (Fig. 5B, left panel). Incubation of these naïve T cells with 50 µM of Nec-1 did not further reduce the cell viability (Fig. 5B, right panel). Thus, the inhibitory effect of Nec-1 on T cell proliferation was not due to toxicity of the inhibitor.

We next measured T cell proliferation by labeling cells with the fluorescent dye CellTracer Violet. CellTracer Violet partitions equally between daughter cells as the parent cell undergoes cell division. Thus, the reduction in fluorescence signal indicates the number of cell divisions the cells have undergone. Treatment of purified murine CD3^+^ T cells with plate-bound anti-CD3 antibody, which mimics TCR signaling, and the costimulatory molecule CD28 potently stimulated cell proliferation as shown by the dilution of the CellTracer Violet dye (Fig. 5C, panel a). At doses that are commonly used in other studies (30 µM), Nec-1 severely impeded cell division (Fig. 5C, compare panels a and e). In fact, cell division was completely abrogated by 50 µM of Nec-1 (Fig. 5C, panel f). Importantly, lower doses of Nec-1 (1 µM) that were sufficient to inhibit FADD^−/−^ T cell necrosis had little effect on T cell division (Fig. 5C, panel b). In addition to cell proliferation, T cell activation also leads to increase in cell size, which can be measured as an increase in forward light scatter by flow cytometry. Consistent with the cell proliferation results, Nec-1 inhibited the formation of “T cell blasts” in response to TCR/CD28 stimulation in a dose-dependent manner (Fig. 5D).

RIP1^−/−^ T cells exhibit a modest defect in T cell proliferation [32]. Thus, the effect of Nec-1 on T cell proliferation could be due to inhibition of RIP1. To evaluate this possibility, we examined the effect of Nec-1 on FADD^−/−^RIP1^−/−^ double knock-out (DKO) T cells. Unlike the FADD^−/−^ or RIP1^−/−^ T cells, the DKO T cells exhibited normal T cell proliferation [10]. We found that proliferation of DKO T cells was similarly inhibited by high doses of Nec-1 (Fig. 5E). Taken together, these results indicate that Nec-1 inhibits T cell proliferation independent of RIP1.

**Figure 6:**
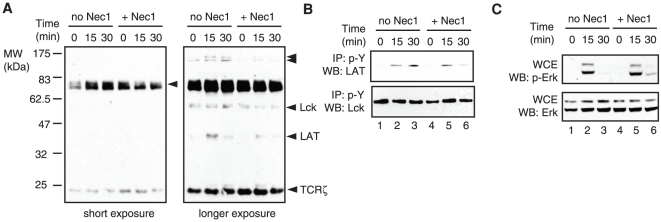
Nec-1 blocks sustained phosphorylation of LAT during T cell activation. (A) Whole cell extracts (WCE) were examined from T cells stimulated with anti-CD3 antibody in the presence or absence of 50 µM Nec-1. Western blot was performed with phospho-tyrosine specific antibody. Short and long exposures were shown to highlight the different phosphorylated species. (B) Nec-1 impairs TCR-induced LAT phosphorylation. Total lysates from activated T cells were subjected to immunoprecipitation with phospho-tyrosine antibody. The resulting immune complex was evaluated for presence of LAT and Lck by Western blot. (C) Nec-1 does not affect Erk phosphorylation. Purified T cells were stimulated as in (A). Western blot was performed with phospo-Erk or total Erk specific antibodies.

The effect of Nec-1 on T cell proliferation suggests that it might target proximal signaling of the TCR. A signature of proximal TCR signaling is the rapid phosphorylation at tyrosine residues for many adaptor proteins and kinases. Western blot with phospho-tyrosine antibody showed that the phosphorylation of several protein species was differentially affected by Nec-1. In particular, the TCR-induced phosphorylation of a 30–40 kDa species that resembled the size of the essential adaptor LAT (linker for activation of T cells) was visibly reduced by Nec-1 (Fig. 6A, right panel). To confirm the effect of Nec-1 on LAT phosphorylation, we immunoprecipitated the T cell lysates with the phospho-tyrosine antibody, followed by Western blot analysis of LAT. We found that while Nec-1 did not dramatically affect the initial phosphorylation of LAT, it abolished sustained LAT phosphorylation (Fig. 6B, compare lanes 3 and 6). In contrast, phosphorylation of Lck, an upstream tyrosine kinase that is phosphorylated at the inhibitory tyrosine Y505 before TCR activation and Y394 after TCR stimulation [33], was not affected by Nec-1 (Fig. 6B). Furthermore, phosphorylation of Erk was not diminished by Nec-1 (Fig. 6C). Thus, we conclude that the abrogation of LAT phosphorylation is one of the possible mechanisms by which Nec-1 inhibits T cell proliferation.

## Discussion

The emerging role of RIP1 and RIP3-dependent necrosis in many disease pathologies have led to the wide use of Nec-1 and its derivatives by many investigators. Nec-1 was thought to specifically inhibit the pro-necrotic kinase function of RIP1 [15], [18] without affecting the kinase activity of RIP3 [6]. In this report, we show that Nec-1 exhibits an unexpected anti-necrotic effect that is independent of RIP1. In L929 cells, necrosis could be induced by treatment with zVAD-fmk or exogenous TNF. However, our data indicated that zVAD-fmk induced necrosis, but not TNF-induced necrosis, was dependent on RIP1. This is surprising because a recent report shows that zVAD-fmk triggers autocrine TNF production that in turn activates necrosis downstream of TNFR1 [25]. The requirement for RIP1 in zVAD-fmk induced necrosis might be explained by its effect on MAP kinase activation [29]. Furthermore, a recent report shows that zVAD-fmk activated a PKC-MAPK signaling axis that is responsible for autocrine production of TNF in L929 cells [25]. Thus, we speculate that the requirement for RIP1 in zVAD-fmk induced necrosis might be attributed to its role in MAP kinase induced TNF production.

In addition to TNF-induced necrosis in L929 cells, it is noteworthy that RIP1-independent but RIP3-dependent necrosis has also been observed in murine cytomegalovirus (MCMV) infection [34]. Therefore, although RIP1 is the upstream activator of RIP3 in most cases, necrosis can proceed in a RIP1-independent manner. Whether RIP3 acts alone or in concert with another RIP1-like factor in these situations will be the subject of further investigation. Importantly, the death of L929 cells with knocked-down expression of RIP1 was inhibited by Nec-1, but not zVAD-fmk. This result rules out the possibility of conversion of necrosis to apoptosis upon RIP1 silencing. While these results could be explained by incomplete silencing of RIP1, it is unlikely because zVAD-fmk induced necrosis was efficiently inhibited in the same cells. Interestingly, residual death could be detected in RIP1-silenced L929 cells treated with TNF, zVAD-fmk and Nec-1. This result suggests the possibility that caspase- and RIP1-independent cell death might occur when both apoptosis and necrosis pathways are inhibited.

Nec-1 modestly reduced expression of PKA-C§, but had little effects on Jnk or Erk activation. The effect of Nec-1 on PKA-C§ is intriguing because PKA-C§ was identified as a putative necrosis mediator previously [6], [35]. Based on our findings, the anti-necrosis effect of Nec-1 previously attributed to RIP1 might have to be re-evaluated. Interestingly, low doses of Nec-1 (1 µM) were sufficient to rescue necrosis in FADD^−/−^ T cells. In contrast, higher doses of Nec-1 (20 µM) were required to fully rescue TNF-induced necrosis in L929 cells. The molecular basis for this differential sensitivity to Nec-1 is unknown, but could be due to different RIP1 expression level or Nec-1 inhibition of other necrosis mediators in L929 cells.

Besides its effect on necrosis, Nec-1 also interferes with proximal TCR signaling. Importantly, the inhibition of TCR signaling and proliferation was only apparent at higher concentrations of Nec-1 that were commonly used in studies assessing the role of RIP1-dependent necrosis in different cells [9], [23]. Nec-1 mediated inhibition of T cell proliferation was not reported in these studies. However, we have observed similar inhibition of T cell proliferation by Nec-1 using different batches of Nec-1 from different vendors (data not shown). Since high dose of Nec-1 had no effect on cell viability, the inhibitory effect of Nec-1 could not be attributed to drug toxicity. Because the full effect of Nec-1 on T cell proliferation required pre-incubation of cells for 1 hour, the discrepant results could be attributed to subtle differences in culture conditions used in the different studies.

Nec-1 blunted sustained phosphorylation of the essential TCR signal adaptor LAT. Interestingly, the initial activation of LAT and the downstream kinase Erk was not affected. Although it is speculative, our results suggest that Nec-1 might stimulate the dephosphorylation of LAT.

In summary, our results highlight the fact that while it is a powerful inhibitor of RIP1, Nec-1 could interfere with the functions of other signaling proteins. We suggest that the dosage of Nec-1 should be carefully titrated in each cell type to determine the minimal dose required for inhibition of necrosis to minimize off-target effects. These results also suggest that results obtained using Nec-1 should be independently confirmed using other approaches such as RNA interference-mediated gene silencing.

## References

[1] Moquin D, Chan FK (2010). The molecular regulation of programmed necrotic cell injury.. Trends Biochem Sci.

[2] Vandenabeele P, Galluzzi L, Vanden Berghe T, Kroemer G (2010). Molecular mechanisms of necroptosis: an ordered cellular explosion.. Nat Rev Mol Cell Biol.

[3] Christofferson DE, Yuan J (2010). Necroptosis as an alternative form of programmed cell death.. Curr Opin Cell Biol.

[4] Challa S, Chan FK (2010). Going up in flames: necrotic cell injury and inflammatory diseases.. Cell Mol Life Sci.

[5] Kono H, Rock KL (2008). How dying cells alert the immune system to danger.. Nat Rev Immunol.

[6] Cho YS, Challa S, Moquin D, Genga R, Ray TD (2009). Phosphorylation-driven assembly of the RIP1-RIP3 complex regulates programmed necrosis and virus-induced inflammation.. Cell.

[7] He S, Wang L, Miao L, Du F, Zhao L (2009). Receptor Interacting Protein Kinase-3 Determines Cellular Necrotic Response to TNF-α.. Cell.

[8] Zhang DW, Shao J, Lin J, Zhang N, Lu BJ (2009). RIP3, an Energy Metabolism Regulator that Switches TNF-Induced Cell Death from Apoptosis to Necrosis.. Science.

[9] Ch'en IL, Beisner DR, Degterev A, Lynch C, Yuan J (2008). Antigen-mediated T cell expansion regulated by parallel pathways of death.. Proc Natl Acad Sci U S A.

[10] Zhang H, Zhou X, McQuade T, Li J, Chan FK (2011). Functional complementation between FADD and RIP1 in embryos and lymphocytes.. Nature.

[11] Kaiser WJ, Upton JW, Long AB, Livingston-Rosanoff D, Daley-Bauer LP (2011). RIP3 mediates the embryonic lethality of caspase-8-deficient mice.. Nature.

[12] Oberst A, Dillon CP, Weinlich R, McCormick LL, Fitzgerald P (2011). Catalytic activity of the caspase-8-FLIP(L) complex inhibits RIPK3-dependent necrosis.. Nature.

[13] Ch'en IL, Tsau JS, Molkentin JD, Komatsu M, Hedrick SM (2011). Mechanisms of necroptosis in T cells.. J Exp Med.

[14] Trichonas G, Murakami Y, Thanos A, Morizane Y, Kayama M (2010). Receptor interacting protein kinases mediate retinal detachment-induced photoreceptor necrosis and compensate for inhibition of apoptosis.. Proc Natl Acad Sci U S A.

[15] Degterev A, Huang Z, Boyce M, Li Y, Jagtap P (2005). Chemical inhibitor of nonapoptotic cell death with therapeutic potential for ischemic brain injury.. Nat Chem Biol.

[16] You Z, Savitz SI, Yang J, Degterev A, Yuan J (2008). Necrostatin-1 reduces histopathology and improves functional outcome after controlled cortical impact in mice.. J Cereb Blood Flow Metab.

[17] Smith CC, Davidson SM, Lim SY, Simpkin JC, Hothersall JS (2007). Necrostatin: a potentially novel cardioprotective agent?. Cardiovasc Drugs Ther.

[18] Degterev A, Hitomi J, Germscheid M, Ch'en IL, Korkina O (2008). Identification of RIP1 kinase as a specific cellular target of necrostatins.. Nat Chem Biol.

[19] Chan FK, Lenardo MJ (2000). A crucial role for p80 TNF-R2 in amplifying p60 TNF-R1 apoptosis signals in T lymphocytes.. Eur J Immunol.

[20] Chan FK, Shisler J, Bixby JG, Felices M, Zheng L (2003). A role for tumor necrosis factor receptor-2 and receptor-interacting protein in programmed necrosis and antiviral responses.. J Biol Chem.

[21] Zhang Y, Rosenberg S, Wang H, Imtiyaz HZ, Hou YJ (2005). Conditional Fas-associated death domain protein (FADD): GFP knockout mice reveal FADD is dispensable in thymic development but essential in peripheral T cell homeostasis.. J Immunol.

[22] Vercammen D, Vandenabeele P, Beyaert R, Declercq W, Fiers W (1997). Tumour necrosis factor-induced necrosis versus anti-Fas-induced apoptosis in L929 cells.. Cytokine.

[23] Osborn SL, Diehl G, Han SJ, Xue L, Kurd N (2010). Fas-associated death domain (FADD) is a negative regulator of T-cell receptor-mediated necroptosis.. Proc Natl Acad Sci U S A.

[24] Yu L, Alva A, Su H, Dutt P, Freundt E (2004). Regulation of an ATG7-beclin 1 program of autophagic cell death by caspase-8.. Science.

[25] Wu YT, Tan HL, Huang Q, Sun XJ, Zhu X (2011). zVAD-induced necroptosis in L929 cells depends on autocrine production of TNFalpha mediated by the PKC-MAPKs-AP-1 pathway.. Cell Death Differ.

[26] Hitomi J, Christofferson DE, Ng A, Yao J, Degterev A (2008). Identification of a molecular signaling network that regulates a cellular necrotic cell death pathway.. Cell.

[27] Zheng L, Bidere N, Staudt D, Cubre A, Orenstein J (2006). Competitive control of independent programs of tumor necrosis factor receptor-induced cell death by TRADD and RIP1.. Mol Cell Biol.

[28] Vanlangenakker N, Vanden Berghe T, Bogaert P, Laukens B, Zobel K (2011). cIAP1 and TAK1 protect cells from TNF-induced necrosis by preventing RIP1/RIP3-dependent reactive oxygen species production.. Cell Death Differ.

[29] Devin A, Lin Y, Liu ZG (2003). The role of the death-domain kinase RIP in tumour-necrosis-factor-induced activation of mitogen-activated protein kinases.. EMBO Rep.

[30] Biton S, Ashkenazi A (2011). NEMO and RIP1 control cell fate in response to extensive DNA damage via TNF-alpha feedforward signaling.. Cell.

[31] Zhang J, Cado D, Chen A, Kabra NH, Winoto A (1998). Fas-mediated apoptosis and activation-induced T-cell proliferation are defective in mice lacking FADD/Mort1.. Nature.

[32] Cusson N, Oikemus S, Kilpatrick ED, Cunningham L, Kelliher M (2002). The death domain kinase RIP protects thymocytes from tumor necrosis factor receptor type 2-induced cell death.. J Exp Med.

[33] Wang H, Zeng X, Fan Z, Lim B (2011). RhoH modulates pre-TCR and TCR signalling by regulating LCK.. Cell Signal.

[34] Upton JW, Kaiser WJ, Mocarski ES (2010). Virus inhibition of RIP3-dependent necrosis.. Cell Host Microbe.

[35] Cho Y, Challa S, Chan FK (2011). A RNA interference screen identifies RIP3 as an essential inducer of TNF-induced programmed necrosis.. Adv Exp Med Biol.

[36] Clancy L, Mruk K, Archer K, Woelfel M, Mongkolsapaya J (2005). Preligand assembly domain-mediated ligand-independent association between TRAIL receptor 4 (TR4) and TR2 regulates TRAIL-induced apoptosis.. Proc Natl Acad Sci U S A.

